# Integrated Genomic and Metabolomic Approach to the Discovery of Potential Anti-Quorum Sensing Natural Products from Microbes Associated with Marine Samples from Singapore

**DOI:** 10.3390/md17010072

**Published:** 2019-01-21

**Authors:** Ji Fa Marshall Ong, Hui Chin Goh, Swee Cheng Lim, Li Mei Pang, Joyce Seow Fong Chin, Koh Siang Tan, Zhao-Xun Liang, Liang Yang, Evgenia Glukhov, William H. Gerwick, Lik Tong Tan

**Affiliations:** 1 Natural Sciences and Science Education, National Institute of Education, Nanyang Technological University, 1 Nanyang Walk, Singapore 637616, Singapore; marshall.ong@nie.edu.sg; 2 p53 Laboratory, Agency for Science, Technology and Research (A*STAR), #06-06, Immunos, 8A Biomedical Grove, Singapore 138648, Singapore; hcgoh@p53lab.a-star.edu.sg; 3 St John’s Island National Marine Laboratory, Tropical Marine Science Institute, National University of Singapore, 18 Kent Ridge Road, Singapore 119227, Singapore; sweecheng@nus.edu.sg (S.C.L.); tmstanks@nus.edu.sg (K.S.T.); 4 School of Biological Sciences, Nanyang Technological University, 60 Nanyang Drive, Singapore 637551, Singapore; pang0165@e.ntu.edu.sg (L.M.P.); zxliang@ntu.edu.sg (Z.-X.L.); 5 Singapore Centre for Environmental Life Sciences Engineering, Nanyang Technological University, 60 Nanyang Drive, Singapore 637551, Singapore; chinsf@ntu.edu.sg (J.S.F.C.); yangliang@ntu.edu.sg (L.Y.); 6 Center for Marine Biotechnology and Biomedicine, Scripps Institution of Oceanography, University of California San Diego, La Jolla, CA 92093, USA; eglukhov@ucsd.edu (E.G.); wgerwick@ucsd.edu (W.H.G.)

**Keywords:** marine bacteria, marine sponges, anti-quorum sensing, molecular network, biosynthetic gene clusters

## Abstract

With 70% of the Earth’s surface covered in water, the marine ecosystem offers immense opportunities for drug discovery and development. Due to the decreasing rate of novel natural product discovery from terrestrial sources in recent years, many researchers are beginning to look seaward for breakthroughs in new therapeutic agents. As part of an ongoing marine drug discovery programme in Singapore, an integrated approach of combining metabolomic and genomic techniques were initiated for uncovering novel anti-quorum sensing molecules from bacteria associated with subtidal samples collected in the Singapore Strait. Based on the culture-dependent method, a total of 102 marine bacteria strains were isolated and the identities of selected strains were established based on their 16S rRNA gene sequences. About 5% of the marine bacterial organic extracts showed quorum sensing inhibitory (QSI) activity in a dose-dependent manner based on the *Pseudomonas aeruginosa* QS reporter system. In addition, the extracts were subjected to mass spectrometry-based molecular networking and the genome of selected strains were analysed for known as well as new biosynthetic gene clusters. This study revealed that using integrated techniques, coupled with biological assays, can provide an effective and rapid prioritization of marine bacterial strains for downstream large-scale culturing for the purpose of isolation and structural elucidation of novel bioactive compounds.

## 1. Introduction

The National Institutes of Health (NIH) reported an estimated 17 million infections arising in the U.S. each year resulting in half a million fatalities. Some 65% of these bacterial infections occurring in the human body are biofilm-related [1]. Moreover, pathogenic bacteria in biofilms are found to be more resistant to antibiotics as compared to their planktonic forms [2]. As such, there is an urgent need for new antibacterial treatments. A paradigm shift has been proposed for the development of new antimicrobial drugs that possess antipathogenic and antivirulence activities [3]. Because this strategy requires no killing of bacterial cells, it is proposed to reduce the occurrence of resistant strains [4]. Furthermore, this approach prevents bacterial diseases by attenuating expression of the genes responsible for pathogenicity, such as bacterial attachment, biofilm formation, chemical signaling, evasion of the host immune system, and the secretion of toxins. One such effective strategy proposed is through the interference of pathogenic bacterial quorum sensing systems using natural products [1,5,6,7].

Marine habitats are a tremendous resource for novel therapeutic agents [8]. In particular, microbes isolated from marine-derived samples, including invertebrates and sediments, are known to produce novel bioactive compounds with biomedical potential, such as anticancer, antifungal, antibiotic, and anti-quorum sensing activities [9,10,11,12]. Certain groups of marine invertebrates, such as sponges and corals, are known to harbor a high diversity of microorganisms, including bacteria, Archaea, microalgae, and fungi, which can account for nearly half of the host biomass [13]. For instance, more than 25 bacterial phyla, such as *Acidobacteria*, *Actinobacteria*, *Bacteroidetes*, *Chloroflexi*, *Firmicutes*, *Proteobacteria*, *Spirochaetes*, and *Verrucomicrobia*, just to name a few, have been reported from marine sponges and these microbes represent an attractive sources of natural products in drug discovery efforts [14,15,16]. Moreover, there is also growing evidence that the invertebrate-associated microbes are the true metabolic sources of these marine natural products that were originally reported from the hosts [17,18].

More than 35 years ago, bacterial communication was not widely accepted by microbiologists as they often considered each bacterial cell to have behaved independently from other bacteria. The term quorum sensing only become widespread when Professor Greenberg [19] elucidated the molecular mechanism process where bacteria communicate with each other. This communication mechanism offered ways to interfere with bacteria pathogens and to modulate the microbiome for health applications [19]. Today, targeting the bacterial communication became the forefront in healthcare researches, especially with the worldwide increasing rate of antibiotic resistance gained by the pathogens [20,21,22]. To facilitate our discovery efforts for anti-quorum sensing compounds from bacteria associated with marine samples, we have adopted an integrated-omic approach in this study. Using a culture-dependent method, marine bacteria were isolated from subtidal samples, including sponges and sediments, collected using a rectangular dredge in the Singapore Strait. Initial organic crude extracts prepared via small scale culturing of isolated marine bacterial colonies were screened using the *Pseudomonas aeruginosa* reporter strain for anti-quorum sensing activity. Bacterial extracts with quorum sensing inhibitory (QSI) activity were then analyzed using a mass-spectrometry based metabolomics Global Natural Products Social Molecular Networking platform (GNPS; https://gnps.ucsd.edu/) for compound dereplication [23]. In addition, marine bacterial strains that showed biological activity were subjected to whole genome sequencing for annotation of biosynthetic gene clusters using an antiSMASH bioinformatics tool. The integration of both metabolomics and genomic techniques employed in this study is an effective and informed decision-making approach for the selection of marine bacterial strains with high probability of discovering novel bioactive compounds.

## 2. Results and Discussion

### 2.1. Isolated Microbes Associated with Deep Water Marine Samples

A total of 13 marine samples (see Supplementary Information Table S1), including 10 taxonomically distinct marine sponges and three sediment samples, were collected from the seabed surface using a rectangular dredge in the Singapore Strait (Latitude 01°10′391” N/Longitude 103°45′729” E). The sponge samples were identified (the morphological characters of these sponges were examined under light microscope and scanning electron microscope) as *Xestospongia testudinaria* (01), *Halichondria* sp. (02), *Rhabdastrella globostellata* (03), *Stelletta* sp. (04), *Geodia* sp. (05), *Dysidea* sp. (06), *Coelocarteria singaporensis* (07), *Haliclona* sp. (08), cf. *Leiodermatium* sp. (09), and *Ircinia* sp. (10).

Homogenates from the 13 marine samples were prepared and plated on eight different marine media (see Supplementary Information Table S2) selected based on previous publications on similar isolation work [24,25,26,27]. Colonies displaying interesting morphology, such as bright colors, matte textures, or unique colony shapes, were identified as our colonies of interest. Some of the other colonies commonly appearing across the different isolation agar plates were also isolated as part of the colonies of interest to ensure that we are not bias in our colonies selection for the drug discovery process. This resulted in a total of 102 bacterial colonies of interest (see Supplementary Information Figure S1) obtained over a period of two months’ incubation. The combination of using low nutrient marine media [25], such as A3, A4HT, and A5, coupled with the prolonged incubation period had facilitated the isolation process in our study. The use of minimal nutrient media also aims to mimic a more environmentally relevant culture condition. Such isolation techniques were employed successfully by other researchers to cultivate taxonomically diverse marine bacteria [25]. In summary, samples from *Geodia* sp. yielded the highest number of bacterial isolates (20), followed by sediment sample 11 (18), *Stelletta* sp. (13), *Coelocarteria singaporensis* (11), sediment sample 12 (10), *Haliclona* sp. (9), *Dysidea* sp. (8), *Xestospongia testudinaria* (7), *Rhabdastrella globostellata* and cf. *Leiodermatium* sp. (2), *Halichondria* sp., and *Ircinia* sp. (1). There were no bacteria of interest isolated from sediment sample 13 although there were many fast growing bacteria observed growing on the different isolation agars.

**Figure 1:**
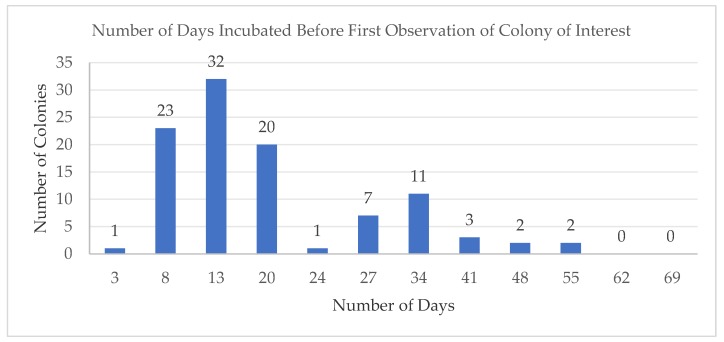
Number of days incubated before first observation of colony appearing on their respective isolation agar. Marine bacterial colonies of interest were tabulated together across all the different types of isolation marine agar on the documented day.

**Figure 2:**
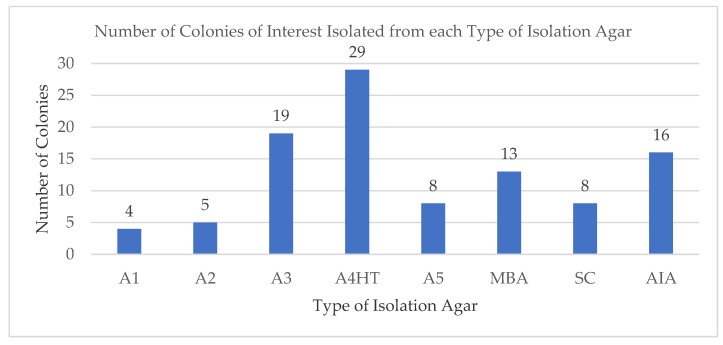
Number of marine bacterial colonies of interest isolated from each type of marine agar used in the study.

The colonies of interest were first documented on day 3 (1 colony) up to a peak at day 13 (32 colonies). As there were no colonies of interest observed beyond day 55, the isolation process was terminated on day 65 (Figure 1). The prolonged duration resulted in a number of rare bacterial types, such as *Kiloniella* sp. and bacterial strains belonging to actinomycetes, which were observed only after 20 days incubation. The typical isolation period carried out by other similar studies were kept to a maximum of 14 days. However, by using low-nutrient media coupled with extended incubation periods, we were able to cultivate additional, previously uncultured marine bacterial taxa. These bacterial colonies were generally observed on the isolation agar plates after between 3–8 weeks of incubation as seen in the second peak centered at day 35 (Figure 2). Towards the end of the monitoring period, many more colonies were recovered, clearly illustrating that the extended incubation time is required for colony formation [25].

It is well known that only about 1% of the bacteria associated with marine samples can be successfully isolated and cultured in the laboratory [28]. Therefore, the choice of appropriate isolation marine media is considered to be of utmost importance at the onset of the study. The eight isolation marine media used in this study consisted of nutrient rich as well as minimal nutrient media types, previously used by other studies [24,25,26,27]. The marine medium, A4HT, a minimal nutrient medium, was observed to produce the highest number of colonies of interest (29 isolates). In contrast, the medium A1, a nutrient rich medium, produced only four colonies of interest (Figure 2). One such explanation could be due to the nutrient rich media that promotes the fast-growing type of bacteria to outgrow the slower-growing colonies in the isolation agar. Although the nutrient rich media yielded far more bacteria colonies than the minimal nutrient media (Figure 2), majority of these fast-growing bacteria were not of interest for isolation, as we our focus was on the slower growing bacteria which are often rare and harder to isolate.

### 2.2. Anti-Quorum Sensing Activity of Marine Bacterial Extracts

**Figure 3:**
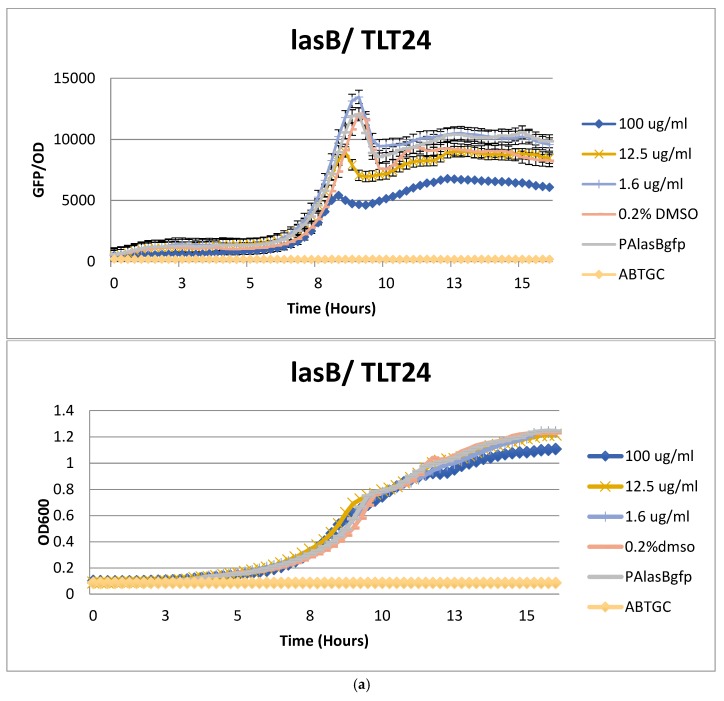

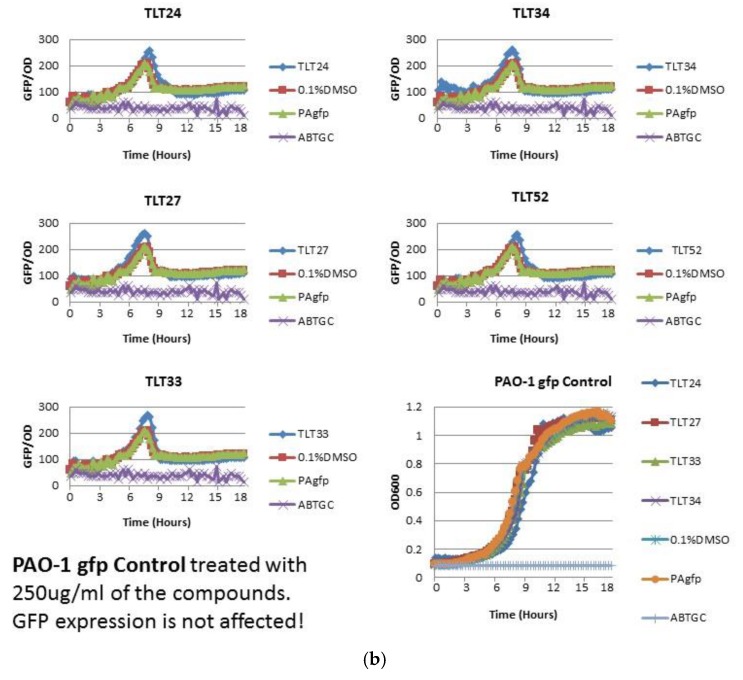
(**a**) The dose-dependent inhibition curves (top graph) from the *Pseudomonas aeruginosa* quorum sensing inhibition bioassay result incubated with crude extract prepared from TLT/SS/14FEB2017/005/A4HT-01/001 (#24) at the various concentration. The growth rates (bottom graph) of the tested strain were not affected, showing that the inhibition effect observed is not due to any death of the tested strain. The experiments were conducted in triplicate and the average reading presented. (**b**) PAO1-*gfp*, tested on all five extracts, showed no reduction in the fluorescence output signals in the control.

With 102 colonies of interest, their 16S rRNA gene sequencing identity not obtained at the time of isolation, isolated from 13 marine samples, their organic extracts were prepared via small scale culturing and screened against the *Pseudomonas aeruginosa* reporter strain, PAO1-*lasB*-*gfp*, [29] for quorum sensing inhibitory activity at 50 mg/mL. Any extract that showed a reduction in the *lasB-gfp* expression in relation to the controls were considered as a positive result. This approach was also adopted by another study for the isolation of disulfide bond-containing ajoene analogues as novel quorum sensing inhibitors of *Pseudomonas aeruginosa* [30]. It is well known that many bacteria species, including pathogenic strains, use quorum-sensing system to coordinate virulence and biofilm development [20]. The reporter strain, *P. aeruginosa*, utilizes at least two *luxI-luxR* homologous QS systems, *las* and *rhl*, to control expression of virulence factors, including elastase, proteases, rhamnolipids, pyocyanin, and cyanide [20]. From our screening efforts, only five marine bacterial extracts, namely TLT/SS/14FEB2017/005/A4HT-01/001 (#24), TLT/SS/14FEB2017/005/A5-01/001 (#27), TLT/SS/14FEB2017/005/MBA-02/004 (#33), TLT/SS/14FEB2017/005/SC-01/001 (#34), and TLT/SS/14FEB2017/007/AIA-02/001 (#52), were found to inhibit the *lasB-gfp* expression. They were further tested to confirm the reduction of the *lasB-gfp* expression in a dose dependent manner without affecting the growth rates of the tested strain. Figure 3a and Supplementary Information Figure S2 illustrates strain TLT/SS/14FEB2017/005/A4HT-01/001 (#24) as a typical example of the extract’s anti-quorum sensing activity result (see Supplementary Information Figures S3 to S6 for the other four extracts’ activity result). Of the five extracts, four were prepared from the marine bacteria strains, namely TLT/SS/14FEB2017/005/A4HT-01/001 (#24), TLT/SS/14FEB2017/005/A5-01/001 (#27), TLT/SS/14FEB2017/005/MBA-02/004 (#33), TLT/SS/14FEB2017/005/SC-01/001 (#34) which were isolated from the sponge sample; *Geodia* sp. (05) and one extract was prepared from bacterial strain (TLT/SS/14FEB2017/007/AIA-02/001) isolated from another sponge sample; *Coelocarteria singaporensis* (07). The induction of the *lasB* gene, encoding elastase, is a good indicator for LasR activity and any decrease in the green fluorescence protein, GFP, would indicate the presence of an antagonist of the 3-oxo-C12-HSL, which inhibits the expression of *lasB*. The GFP expression presented was normalized by dividing its value with the growth measured at their respective time points. The five extracts were further tested with the PAO1-*pqsA-gfp* and PAO1-*rhlA*-*gfp* bioreporters to confirm on their quorum sensing inhibition (see Supplementary Information Figures S2 to S6). The extracts were also tested with PAO1-*gfp* as a control to ensure the authenticity of the targeted corresponding quorum sensing genes instead of the *gfp* itself. All five bacterial extracts did not show any reduction in the fluorescence output signals in the control (Figure 3b). A media blank containing the organic extract of the growth media only was also tested to ensure that there are no compounds from the media which may be responsible for the anti-quorum sensing activity observed giving a false positive result.

### 2.3. MS-Based Molecular Networking of Organic Extracts Derived from Selected Marine Bacterial Strains

**Figure 4:**
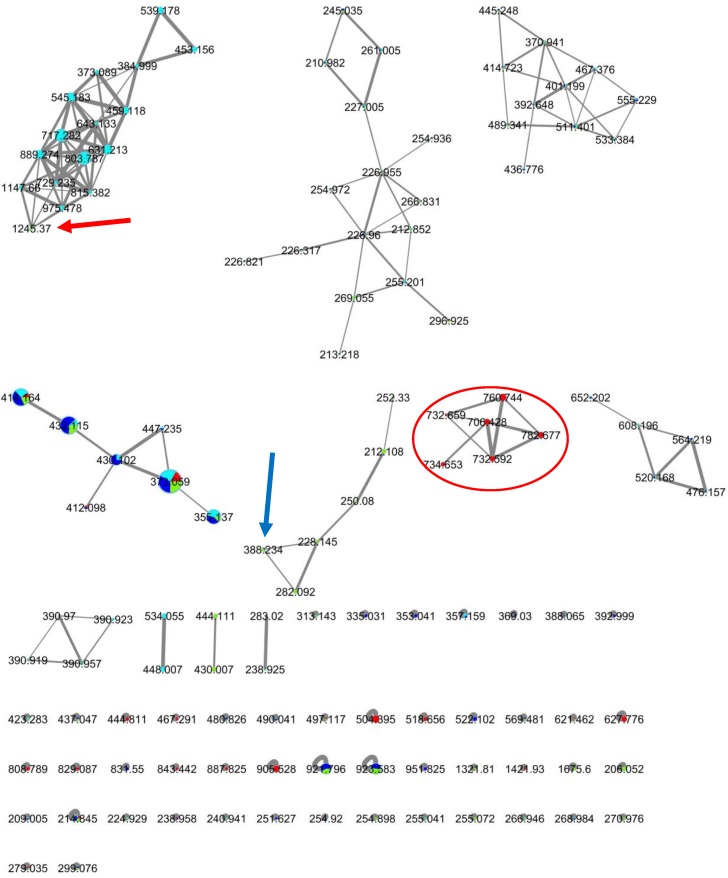
Molecular network of the clusters corresponding to ionizable compounds from marine bacterial extracts TLT/SS/14FEB2017/005/A4HT-01/001 (#24), TLT/SS/14FEB2017/005/SC-01/001 (#34) and TLT/SS/14FEB2017/007/AIA-02/001 (#52). The red nodes represent ions detected from the growth medium, while the cyan, blue and green nodes represent ions detected from the crude extracts #24, #34, and #52, respectively. #24 node cluster showing a single node (red arrow) from #52 (*m*/*z* 1245.37) edging to the cluster with more than 70% similarity. #52 node cluster showing a single node (blue arrow) from #34 and #52 (*m*/*z* 388.234) edging to the cluster with more than 70% similarity. Node cluster (red circle) showing compounds primarily derived from the blank media extract.

From the five bacterial extracts that showed anti-quorum sensing activity, we proceeded with their metabolomic analysis using the MS-based molecular networking platform [31]. Unfortunately, two of these bacterial extracts, #27 and #33, did not achieve optimal ionization during the tandem mass spectrometry based on their respective observed total ion chromatogram (TIC) profile. Therefore, we continued with the analysis of the remaining extracts, namely TLT/SS/14FEB2017/005/A4HT-01/001 (#24), TLT/SS/14FEB2017/005/SC-01/001 (#34) and TLT/SS/14FEB2017/007/AIA-02/001 (#52), for compound dereplication and comparative analyses (Figure 4).

This dereplication process would serve as a rapid analytical tool to shortlist the plethora of crude extracts prepared from the culturing of the isolated bacterial strains. Such analysis provided an informed decision approach in prioritizing of selected bacterial for the downstream large-scale culturing and eventual purification process for potential new compounds. However, the dereplication analysis is preliminary in nature as it relies solely on the MS/MS fragmentation pattern of molecules compared against the MS/MS spectra deposited in the GNPS compound library. The library matches would be more accurate if both query and library’s mass spectrometry were performed on a high resolution system. As our study did not employ a HR-MS/MS system for our mass spectrometry, further in-depth analysis has to be performed if we were to confirm the identity of the matched sample compound due to the difference in the mass value.

From the analysis of the dereplication results of the three marine bacterial extracts that exhibited anti-quorum sensing activity, the extracts did not show any matched known compounds within the GNPS database. Therefore, the hypothesis is that we could be dealing with potentially novel or new analogue compounds that are responsible for the anti-quorum sensing activity. From the molecular networking analysis, each of the two bacterial strains, TLT/SS/14FEB2017/005/A4HT-01/001 (#24) and TLT/SS/14FEB2017/007/AIA-02/001 (#52), had produced unique clusters of compounds. The unique cluster from strain #24 showed it contained relatively higher molecular weight compounds than that from strain #52. The strain specificity nature as seen from the molecular network holds much potential for the discovery of bioactive compounds as each strain has its own unique chemistry. The use of this MS-based metabolomic technique coupled together with the biological assays had confirmed our priority selection of the three bacterial strains for the downstream large-scale culturing and further chemical investigations.

### 2.4. Phylogenetic Analysis of Selected Marine Bacterial Strains

In this study, we performed the isolation and purification of genomic material from selected bacterial strains based on their positive quorum sensing inhibitory activity as well as molecular networking data. A number of bacterial strains isolated from sponge, *Geodia* sp., that did not show positive bioassay activity were also included for the phylogenetic analysis as there was a high number of bacterial strains isolated from this sponge species. By subjecting the other bacterial strains that were isolated from this sponge, we hope to have an insight on the sponge bacterial communities. However, we were not able to obtain good quality DNA from the extraction process for a number of the selected bacterial strains. Only 29 strains were eventually identified based on their 16S rRNA gene sequencing (Table 1). It was revealed that these 29 strains were affiliated to 14 different bacterial genera, of which one falls under the uncultured category, after comparing against sequences in the NCBI GenBank database. Two out of the three priority strains that are of particular interest to us, based on positive bioassay activity and molecular networking analysis, exhibited more than 98% confirmation to uncultured [32] bacterial clones (Table 1). The findings suggested that the two bacterial strains probably belong to a putatively novel bacterial genus class or that the marine bacteria strains in question were not previously studied in detail.

The highest number of colonies of interest isolated were found to be affiliated with the genus *Kocuria* (nine colonies), followed by *Micrococcus* and uncultured (three colonies each), *Micromonospora*, *Gordonia*, and *Bacillus* (two colonies each), and the rest each having one colony isolated each (see Supplementary Information Figure S7). The majority of the colonies of interest are classified under the Actinomycetales phylum (*Kocuria*, *Micrococcus*, *Micromonospora*, *Streptomonospora*, *Gordonia*) and the rest are Proteobacteria phylum (*Kiloniella*, *Pseudomonas*, *Alcanivorax*, *Vibrio*) and Firmicutes phylum (*Bacillus*, *Staphylococcus*, *Paenibacillus*).

**Figure 5:**
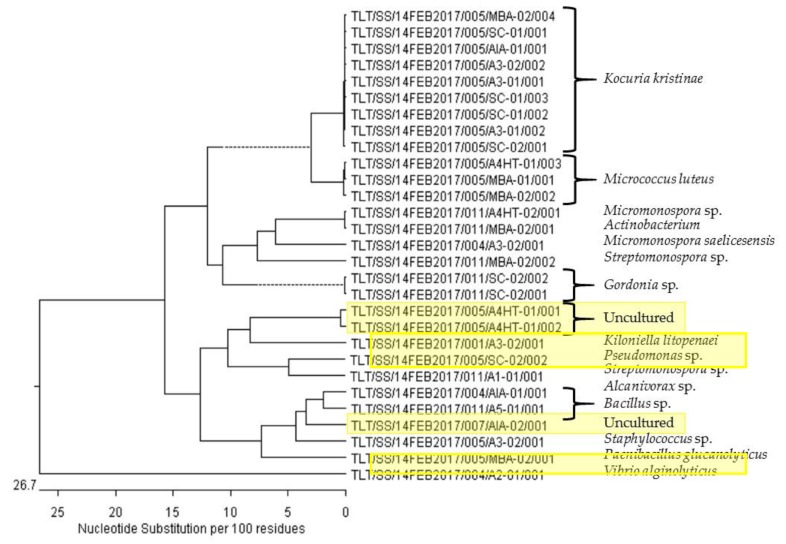
Phylogenetic tree of marine bacteria based on the 16S rRNA gene sequencing result, constructed through DNASTAR^®^ Lasergene MegAlign Pro software Version 15.

The phylogenetic analysis (Figure 5) revealed that the uncultured bacterial clones, TLT/SS/14FEB2017/005/A4HT-01/001 (#24), TLT/SS/14FEB2017/005/A4HT-01/002 (#25) and TLT/SS/14FEB2017/007/AIA-02/001 (#52), form a distinct clade possibly belonging to a novel genus that is closely related with *Kiloniella* and *Bacillus* due to its closest relative proximity. In order to validate the taxonomic position of these three putatively novel bacterial clones isolated in our study, further in-depth phenotypic and genotypic characterization would have to be pursued.

### 2.5. Annotation of Biosynthetic Gene Clusters of Selected Marine Bacterial Genome

In our study, we had presented the whole genome sequencing of two of the priority strains, namely TLT/SS/14FEB2017/005/A4HT-01/001 (#24) and TLT/SS/14FEB2017/007/AIA-02/001 (#52), for annotation of the natural product biosynthetic gene clusters (BGC) using antiSMASH 4.0.2 (Table 2) [33,34]. The whole genome sequencing experiment was conducted by a commercial company, Axil Scientific Pte Ltd., and the results were interpreted in-house. The preliminary analysis conducted will form the basis for future studies into these two putative novel marine bacteria that were shown to produce bioactive compounds having anti-quorum sensing activity. Such an approach was successfully used to discover bioactive compounds, such as the anticancer compound, retimycin A [26], and the antifungal with cytotoxic activity compound, malyngamide C [35].

The complete genome of the two marine bacteria, TLT/SS/14FEB2017/005/A4HT-01/001 (#24) and TLT/SS/14FEB2017/007/AIA-02/001 (#52), were sequenced at the 1st Base Laboratories (Singapore) using 2x150PE format with Miseq Platform. The genome for #24 and #52 were found to be 3.8 Mb and 5.6 Mb in length, with a GC content of 41.3% and 32.5%, respectively. The biosynthetic potential of the bacteria were assessed using antiSMASH 4.0.2 [33,34]. Out of six putative gene clusters identified that are housed in the #24 bacterium genome, there is one siderophore cluster (50% similar to the Carotenoid BGC), one bacteriocin cluster, one terpene cluster, one type III polyketide/saccharide hybrid cluster, one non-ribosomal peptide synthetase gene cluster (85% similar to the Lichenysin BGC), and one others cluster that is 85% similar to the Bacilysin BGC. There are 13 putative gene clusters identified that are housed in the #52 bacterium genome. Of these 13 clusters, there is one siderophore cluster (100% similar to the Petrobactin BGC), four non-ribosomal peptide synthetase gene clusters, one terpene cluster, four bacteriocin clusters, one sactipeptide cluster (100% similar to the Thurincin H BGC), one arylpolyene cluster, and one others cluster.

## 3. Experimental

### 3.1. Sample Collection and Processing

In total, 13 marine samples (10 sponges and three sediments, Figure 1) were collected by mechanical dredging (National Parks Board Permit Number: NP/RP17-007), at a depth of between 35 to 60 m, off the seabed of the Singapore Strait (Latitude 01°10′391” N/Longitude 103°45′729” E) on 14 February 2017. The samples were kept in plastic bags containing seawater and transported to the laboratory as soon as the collection was completed. A 1 cm^3^-sized (for sponge) or 1 g (for sediment) of each sample was removed and cleaned with sterile artificial seawater to remove loosely attached microorganisms that may be present on the sample. They were thoroughly homogenized in a mortar with 10 mL of sterile artificial seawater. The supernatant was heat-shocked at 65 °C for 10 min and subsequently plated out in duplicates onto the isolation agar plates.

### 3.2. Cultivable Microbial Isolation

Eight different types of marine media were used for the isolation of cultivable marine bacteria. All media were sterilized by autoclaving and supplemented with 0.2 µm pore size filtered of the respective concentration of potassium dichromate/cycloheximide and nalidixic acid sodium salt solution to minimize fast growing microorganisms from dominating over the slower growing bacteria strains of interest. Potassium dichromate and cycloheximide inhibit fungal growth and nalidixic acid sodium salt inhibits many fast-growing Gram-negative bacteria [28]. In total, 208 isolation agar plates were used. The plates were incubated at 25 °C for up to a projected duration of three months with weekly observations and isolation of the colonies of interest.

Isolates were picked and re-streaked onto the standardized MBA until visually free of contaminants. They were then inoculated into a 250 mL Erlenmeyer flask containing 100 mL of liquid media (BD Difco^TM^ Marine Broth supplemented with 0.015 g/L nalidixic acid sodium salt, 0.05 g/L potassium dichromate) and incubated at 25 °C with 150 rpm shaking for 14 days. A portion of these liquid culture were supplemented with 20% glycerol and stored at −80 °C for long-term strain archival. All isolates were tagged accordingly (e.g., TLT/SS/14FEB2017/001 (Sample Number)/A1-01 (Type of Agar and Agar Plate Number)/001 (Colony Number on the Agar Plate).

### 3.3. Molecular Identification and Phylogenetic Analysis

Bacteria isolates genomic DNA was extracted from each cell pellet using the Zymo Research Quick-DNA™ fungal/bacterial microprep kit protocol. The PCR amplification of the 16S rRNA gene was performed in 50 µL volumes using the universal primers 27F (5′-GAGTTTGATCCTGGCTCAG-3′) and 1525R (5′-AGAAAGGAGGTGATCCAGCC-3′). Thermocycling parameters consisted of initial denaturation at 95 °C for 5 min, 34 cycles of 95 °C for 10 s, 53 °C for 10 s and 68 °C for 10 min followed by a final extension at 68 °C for 2 min. PCR amplification was evaluated by agarose gel electrophoresis (1% agarose, 1× TBE buffer stained with gel red). 16S rRNA gene amplicons were sequenced by 1st Base Laboratories (Singapore) using the primers 27F and 1525R. Sequences (majority ≈ 1500 base pairs) were analysed using the Basic Local Alignment Search Tool (BLAST), to compare and identify the gene sequences with known bacterial 16S rRNA gene sequences deposited in the GenBank database (http://www.ncbi.nlm.nih.gov/BLAST).

The 16S rRNA gene sequences from the bacteria isolates were analysed using DNASTAR^®^ Lasergene Pro software Version 15. The gene sequences were uploaded onto the NCBI Nucleotide BLAST platform where the query sequences were cross referenced within the NCBI database. Multiple alignment of all 16S rRNA gene sequences was achieved using the DNASTAR^®^ Lasergene MegAlign Pro software Version 15 to construct the phylogenetic tree.

### 3.4. Organic Extract Preparation and Anti-Quorum Sensing Bioassay

Using the liquid-liquid solvent partitioning methodology, an equal volume of ethyl acetate was added to the liquid cultures and mechanically shaken by hand (three times for 30 s each) before leaving the mixture to partition into their respective phases and the ethyl acetate phase was collected. The extraction process was repeated a second time. The collected ethyl acetate phase was dried using a rotary evaporator and stored at −20 °C until required.

The anti-quorum sensing bioassay was carried out against the *Pseudomonas aeruginosa* system. Each of the extracts, conducted in triplicates, were prepared in 96-well microtiter plates at 50 mg/mL stock concentration dissolved in 100% DMSO. The mixtures were then added with ABTGC medium; (see Supplementary Information Table S2) and serial diluted to give a concentration of 100 µg/mL in the first well (with 0.2% of DMSO). An overnight culture of PAO1-*lasB-gfp* strain, grown in Luria-Bertani medium at 37 °C, 200 rpm, was diluted in ABTGC medium to an optical density of 0.02 at OD600 which correspond to 2.5 × 10^7^ CFU/mL. An equal amount of the bacterial suspension was added to the wells to reach a final inhibitor concentration of 10 µM. DMSO control and blank control were used and the microtiter plates were incubated at 37 °C in Tecan Infinate 200 Pro plate reader to measure the cell density (OD600) and green fluorescence protein fluorescence (excitation at 485 nm, emission at 535 nm) with 15 min intervals for up to 18 h. For the *Pseudomonas aeruginosa* Rhl and Pqs inhibition assay, the same methodology was applied (see Table 3 for strains and plasmids information).

### 3.5. Mass Spectrometry-Based Molecular Networking

The tandem mass spectrometry data for the isolates were generated at the Center for Marine Biotechnology and Biomedicine, Scripps Institution of Oceanography, University of California San Diego, USA. The extracts and blank media control were prepared to a concentration of 0.5 mg/mL in methanol solution before injecting 10 µL aliquots into the Thermo Finnigan (San Diego, CA, USA) LCQ Advantage Max mass spectrometer system attached to a Thermo Finnigan (San Diego, CA, USA) Surveyor Autosampler-Plus, a LC-Pump-Plus, and a PDA-Plus system to obtain the mass spectrometry data. The chromatographic analysis was done with a Phenomenex (Torrance, CA, USA) Kinetex C18 100 Å (2.6 µm, 100 × 4.6 mm) column with a 600 µL/min flow and a gradient elution mobile phase of acetonitrile and water with 0.1% formic acid. The program was set to 30% acetonitrile for the first 5 min, 30% to 99% acetonitrile over 19 min and lastly 99% acetonitrile for 3 min before the column was equilibrated back to the starting conditions. The mass spectrometry was carried out in positive ionization mode with a spray voltage of 5 kV and the capillary temperature set to 400 °C mode where the first, second, and third most intense ions of a full scan mass spectrum were subjected to tandem mass spectrometry (MS/MS) analysis. The MS/MS scans were obtained for selected ions with CID fragmentation, and an isolation width *m*/*z* of 2.0. The data files generated by the LC/MS/MS were analyzed using the Thermo Xcalibur software 2.0.6 converted from .raw to .mzXML format using the MSConvert program from ProteoWizard and uploaded onto the GNPS server (http://gnps.ucsd.edu) and the molecular networking performed using the GNPS data analysis workflow employing a special spectral clustering algorithm.

The network spectra and the library reference spectra were required to have a minimum cosine score threshold of 0.7 and a minimum of two matched peaks in order to be considered for spectral library annotation [36] and a minimum of six matched fragment ions. Further edges between two nodes were kept in the network if each of the nodes appeared in each other’s top 10 most similar nodes. The input data were searched against annotated reference spectra of the MS/MS library within GNPS. For the visualization of compounds from the dereplication hits, the results were exported and viewed directly with the pie-chart creating tool (nodeCharts plugin for Cytoscape) within Cytoscape 3.5.1.

## 4. Conclusions

Our study is the first in Singapore to use an integrated biological, genomic and metabolomic approach for the discovery of anti-quorum sensing molecules from subtidal marine samples to the best of our knowledge. We isolated 102 marine bacteria from 13 marine sponge and sediment samples obtained from dredged samples from the seabed of the Singapore Strait. From our findings, five of these isolated marine bacteria produce compounds that have anti-quorum sensing activity based on their bacterial organic extracts. The compounds produced by three of these marine bacteria obtained from the sponges, *Geodia* sp. and *Coelocarteria singaporensis*, are possibly novel or new analogues as they do not have any compound match based on the GNPS library search. The results highlighted the importance of a streamlined and targeted approach for bioprospecting novel bioactive compounds. Molecular networking-based dereplication is a valuable tool for prioritizing microbial isolates for downstream processing and chemical investigation [37]. Such an approach has provided us with at least three marine bacterial isolates for further chemical analysis, including compound isolation and purification via large scale culturing.

## Figures and Tables

**Table 1:** Overview of isolated marine bacterial strains identified using 16S rRNA gene sequencing with analysis done using DNASTAR^®^ Lasergene Seqbuilder Pro Version 15 and NCBI Blastn online platform.

Sample #	Marine Bacterial Code Number	Source	Base Pairs #	Identification (% Similarity)	NCBI Accession #
1	TLT/SS/14FEB2017/001/A3-02/001	*Xestospongia* sp.	1433	*Kiloniella litopenaei* (98)	LT717346
8	TLT/SS/14FEB2017/004/A2-01/001	*Stelletta* sp.	536	*Vibrio alginolyticus* (90)	CP017919
10	TLT/SS/14FEB2017/004/A3-02/001	*Stelletta* sp.	1400	*Micromonospora saelicesensis* (97)	KM37003
16	TLT/SS/14FEB2017/004/AIA-01/001	*Stelletta* sp.	1484	*Bacillus* sp. (99)	KC953600
20	TLT/SS/14FEB2017/005/A3-01/001	*Geodia* sp.	1467	*Kocuria kristinae* (99)	KR230389
21	TLT/SS/14FEB2017/005/A3-01/002	*Geodia* sp.	1467	*Kocuria kristinae* (99)	KR230389
22	TLT/SS/14FEB2017/005/A3-02/001	*Geodia* sp.	1491	*Staphylococcus* sp. (99)	FR839669
23	TLT/SS/14FEB2017/005/A3-02/002	*Geodia* sp.	1466	*Kocuria kristinae* (99)	KR230389
24	TLT/SS/14FEB2017/005/A4HT-01/001	*Geodia* sp.	1398	Uncultured (98)	EF574305
25	TLT/SS/14FEB2017/005/A4HT-01/002	*Geodia* sp.	1384	Uncultured (96)	KJ814073
26	TLT/SS/14FEB2017/005/A4HT-01/003	*Geodia* sp.	1449	*Micrococcus luteus* (99)	KP345957
29	TLT/SS/14FEB2017/005/MBA-01/001	*Geodia* sp.	1450	*Micrococcus luteus* (99)	KT805418
30	TLT/SS/14FEB2017/005/MBA-02/001	*Geodia* sp.	1489	*Paenibacillus glucanolyticus* (99)	CP015286
31	TLT/SS/14FEB2017/005/MBA-02/002	*Geodia* sp.	1447	*Micrococcus luteus* (99)	KF993668
33	TLT/SS/14FEB2017/005/MBA-02/004	*Geodia* sp.	1465	*Kocuria kristinae* (99)	KR230389
34	TLT/SS/14FEB2017/005/SC-01/001	*Geodia* sp.	1468	*Kocuria* kristinae (99)	DQ158132
35	TLT/SS/14FEB2017/005/SC-01/002	*Geodia* sp.	1461	*Kocuria kristinae* (99)	KR230389
36	TLT/SS/14FEB2017/005/SC-01/003	*Geodia* sp.	1455	*Kocuria* kristinae (99)	DQ158132
37	TLT/SS/14FEB2017/005/SC-02/001	*Geodia* sp.	1831	*Kocuria* kristinae (98)	KF075509
38	TLT/SS/14FEB2017/005/SC-02/002	*Geodia* sp.	1475	*Pseudomonas* sp. (99)	KT034415
39	TLT/SS/14FEB2017/005/AIA-01/001	*Geodia* sp.	1466	*Kocuria* sp. (99)	KR230389
52	TLT/SS/14FEB2017/007/AIA-02/001	*Coelocarteria singaporensis*	1481	Uncultured (99)	KX859231
64	TLT/SS/14FEB2017/011/A1-01/001	Sediment	1477	*Alcanivorax* sp. (99)	KU954765
66	TLT/SS/14FEB2017/011/A4HT-02/001	Sediment	1447	*Micromonospora* sp. (99)	AB738798
67	TLT/SS/14FEB2017/011/A5-01/001	Sediment	1486	*Bacillus* sp. (99)	AJ438301
68	TLT/SS/14FEB2017/011/MBA-02/001	Sediment	1445	*Actinobacterium* (99)	JN049491
69	TLT/SS/14FEB2017/011/MBA-02/002	Sediment	1466	*Streptomonospora* sp. (99)	JX007947
70	TLT/SS/14FEB2017/011/SC-02/001	Sediment	1455	*Gordonia* sp. (99)	EU590659
71	TLT/SS/14FEB2017/011/SC-02/002	Sediment	1456	*Gordonia* sp. (99)	CP002907

**Table 2:** Summary of antiSMASH 4.0.2 output from the whole genome sequences obtained for TLT/SS/14FEB2017/005/A4HT-01/001 (#24) with six different clusters identified with different types of secondary metabolites and Summary of antiSMASH 4.0.2 output from the whole genome sequences obtained for TLT/SS/14FEB2017/007/AIA-02/001 (#52) with 13 different clusters identified with different types of secondary metabolites.

**TLT/SS/14FEB2017/005/A4HT-01/001 (#24)**				
**Cluster**	**Type**	**From**	**To**	**Most Similar Known Cluster**
Cluster 1	Other	704,902	746,323	Bacilysin_biosynthetic_gene_cluster
				(85% of genes show similarity)
Cluster 2	Terpene-Siderophore	57,654	91,248	Carotenoid_biosynthetic_gene_cluster
				(50% of genes show similarity)
Cluster 3	Bacteriocin	442,553	452,879	-
Cluster 4	Terpene	56,166	78,121	-
Cluster 5	T3pks	116,521	156,058	-
Cluster 6	Nrps	143,837	227,604	Lichenysin_biosynthetic_gene_cluster
				(85% of genes show similarity)
**TLT/SS/14FEB2017/007/AIA-02/001 (#52)**				
**Cluster**	**Type**	**From**	**To**	**Most Similar Known Cluster**
Cluster 1	Siderophore	387,071	400,778	Petrobactin_biosynthetic_gene_cluster (100% of genes show similarity)
Cluster 2	Nrps	687,959	737,684	Bacillibactin_biosynthetic_gene_cluster (46% of genes show similarity)
Cluster 3	Nrps	57,214	116,594	Polyoxypeptin_biosynthetic_gene_cluster (5% of genes show similarity)
Cluster 4	Terpene	35,609	57,462	Molybdenum_cofactor_biosynthetic_gene_cluster (11% of genes show similarity)
Cluster 5	Other	96,159	139,740	-
Cluster 6	Bacteriocin	156,762	170,627	-
Cluster 7	Nrps	17,996	65,012	-
Cluster 8	Bacteriocin	80,566	90,814	-
Cluster 9	Bacteriocin	3212	13,541	-
Cluster 10	Nrps	117,416	183,324	-
Cluster 11	Sactipeptide	1	17,542	Thurincin_H_biosynthetic_gene_cluster (100% of genes show similarity)
Cluster 12	Arylpolyene-Nrps	46,467	94,641	-
Cluster 13	Bacteriocin	28,116	40,314	-

**Table 3:** Strains and Plasmid Used in This Study [30].

Strains or Plasmids	Relevant Genotype and/or Characteristics
**Strains**	
PAO1	ATCC *Pseudomonas aeruginosa*
PAO1-*gfp*	GFP-tagged wild-type *Pseudomonas aeruginosa*
PAO1-*lasB*-*gfp*	PAO1 containing *lasB-gfp* (ASV) translational reporter fusion
PAO1 ΔlasIΔrhlI	Gentamicin Resistance; PAO1 *lasI* and *rhlI* mutant
**Plasmids**	
P*rhlA-gfp*	Gentamicin Resistance/Carbenicillin Resistance; pUCPNotI-based plasmid carrying RlhRregulated *rhlA-gfp* (ASV) translational fusion
P*pqsA-gfp*	Gentamicin Resistance/Carbenicillin Resistance; pUCP22NotI-based plasmid carrying *pqsA-gfp* (ASV) transcriptional fusion

## Supplementary Materials

The following are available online at http://www.mdpi.com/1660-3397/17/1/72/s1, Table S1: Thirteen deep water marine samples were collected from the seabed on 14 February 2017 using a rectangular dredge, at a depth of between 35 to 60 m in the Singapore Strait (Latitude 01°10′.391 N/Longitude 103°45′.729 E). 01: *Xestospongia testudinaria*, 02: *Halichondria* sp., 03: *Rhabdastrella globostellata*, 04: *Stelletta* sp., 05: *Geodia* sp., 06: *Dysidea* sp., 07: *Coelocarteria singaporensis*, 08: *Haliclona* sp., 09: cf. *Leiodermatium* sp., 10: *Ircinia* sp., 11 to 13 are marine sediments (the morphological characters of these sponge samples were examined under light microscope and scanning electron microscope). Table S2: Composition of the eight media and other reagents used for the isolation of culturable marine bacteria and other bioassays. Figure S1: An example of colonies of interest (red arrow) that were isolated from the eight different isolation media. Colonies displaying interesting morphology, such as bright colors, matte textures, or unique colony shapes, were identified as our colonies of interest. Some of the other colonies (blue arrow) commonly appearing across the different isolation agar plates were also isolated as part of the colonies of interest to ensure that we are not bias in our colonies selection for the drug discovery process. Figure S2 to S6: The dose-dependent inhibition curves (left graphs) from the *Pseudomonas aeruginosa* quorum sensing inhibition bioassay result incubated with crude extract prepared from TLT/SS/14FEB2017/005/A4HT-01/001 (#24) TLT/SS/14FEB2017/005/A5-01/001 (#27), TLT/SS/14FEB2017/005/MBA-02/004 (#33), TLT/SS/14FEB2017/005/SC-01/001 (#34), and TLT/SS/14FEB2017/007/AIA-02/001 (#52) respectively, at the various concentration. The growth rates (right graphs) of the tested strain were not affected, showing that the inhibition effect observed is not due to any death of the tested strain. The experiments were conducted in triplicate and the average reading presented. Figure S7: Number of colonies of interest identified per genera based on the 16S rRNA gene sequencing result.
