# Supplementary material for: Integrated Genomic and Metabolomic Approach to the Discovery of Potential Anti-Quorum Sensing Natural Products from Microbes Associated with Marine Samples from Singapore
Source: Mar Drugs. 2019 Jan 21;17(1):72. doi: 10.3390/md17010072 (PMC6356914; doi:10.3390/md17010072)
Supplement: Supplementary file 1 [file marinedrugs-17-00072-s001.pdf]

Supplementary Information

Integrated Genomic and Metabolomic Approach to the Discovery of Potential Anti-Quorum Sensing Natural Products from Microbes Associated with Marine Samples from Singapore

Ji Fa Marshall Ong [1], Hui Chin Goh [2], Swee Cheng Lim [3], Li Mei Pang [4], Joyce Seow Fong Chin [5], Koh Siang Tan [3], Zhao-Xun Liang [4], Liang Yang [5], Evgenia Glukhov [6], William H. Gerwick [6] and Lik Tong Tan [1,*]

[1] Natural Sciences and Science Education, National Institute of Education, Nanyang Technological University, 1 Nanyang Walk, Singapore 637616, Singapore; marshall.ong@nie.edu.sg

[2] p53 Laboratory, Agency for Science, Technology and Research (A*STAR), #06-06, Immunos, 8A Biomedical Grove, Singapore 138648, Singapore; hcgoh@p53lab.a-star.edu.sg

[3] St John's Island National Marine Laboratory, Tropical Marine Science Institute, National University of Singapore, 18 Kent Ridge Road, Singapore 119227, Singapore; sweecheng@nus.edu.sg (S.C.L.); tmstanks@nus.edu.sg (K.S.T.)

[4] School of Biological Sciences, Nanyang Technological University, 60 Nanyang Drive, Singapore 637551, Singapore; pang0165@e.ntu.edu.sg (L.M.P.); zxliang@ntu.edu.sg (Z.-X.L.)

[5] Singapore Centre for Environmental Life Sciences Engineering, Nanyang Technological University, 60 Nanyang Drive, Singapore 637551, Singapore; chinsf@ntu.edu.sg (J.S.F.C.); yangliang@ntu.edu.sg (L.Y.)

[6] Center for Marine Biotechnology and Biomedicine, Scripps Institution of Oceanography, University of California San Diego, La Jolla, CA 92093, USA; eglukhov@ucsd.edu (E.G.); wgerwick@ucsd.edu (W.H.G.)

* Correspondence: liktong.tan@nie.edu.sg; Tel.: +65-6790-3842

Contents

**Table S1**	Photograph of marine samples collected	Page 3
**Table S2**	Composition of media and other reagents used	Page 4
**Figure S1**	Photograph of colonies of interest	Page 5
**Figure S2 to S6**	Dose-dependent inhibition curves of TLT/SS/14FEB2017/005/A4HT-01/001 (#24), TLT/SS/14FEB2017/005/A5-01/001 (#27), TLT/SS/14FEB2017/005/MBA-02/004 (#33), TLT/SS/14FEB2017/005/SC-01/001 (#34), and TLT/SS/14FEB2017/007/AIA-02/001 (#52) respectively	Page 6 to 13
**Figure S7**	Number of colonies of interest identified per genera based on the 16S rRNA gene sequencing result.	Page 14

Table S1. Thirteen deep water marine samples were collected from the seabed on 14 February 2017 using a rectangular dredge, at a depth of between 35 to 60 m in the Singapore Strait (Latitude 01°10'.391N / Longitude 103°45'.729E). 01: *Xestospongia testudinaria*, 02: *Halichondria* sp., 03: *Rhabdastrella globostellata*, 04: *Stelletta* sp., 05: *Geodia* sp., 06: *Dysidea* sp., 07: *Coelocarteria singaporensis*, 08: *Haliclona* sp., 09: cf. *Leiodermatium* sp., 10: *Ircinia* sp., 11 to 13 are marine sediments (the morphological characters of these sponge samples were examined under light microscope and scanning electron microscope).

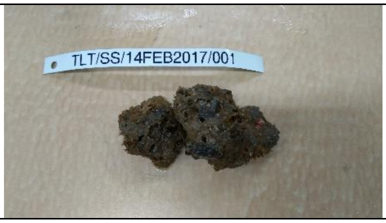

TLT/SS/14FEB2017/001
(01) Sponge

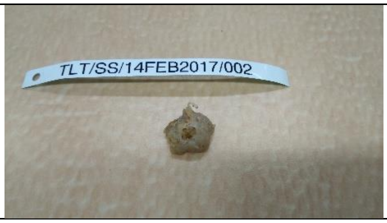

TLT/SS/14FEB2017/002
(02) Sponge

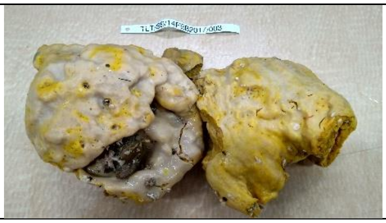

TLT/SS/14FEB2017/003
(03) Sponge

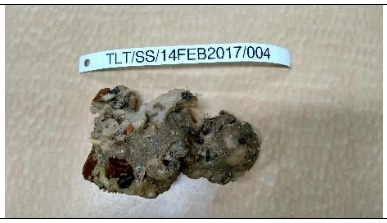

TLT/SS/14FEB2017/004
(04) Sponge

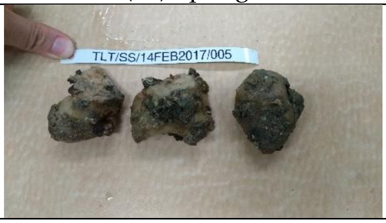

TLT/SS/14FEB2017/005
(05) Sponge

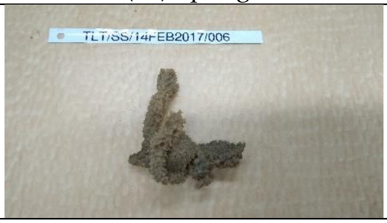

TLT/SS/14FEB2017/006
(06) Sponge

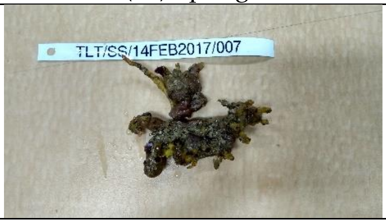

TLT/SS/14FEB2017/007
(07) Sponge

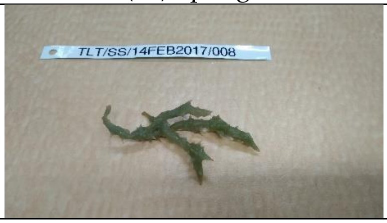

TLT/SS/14FEB2017/008
(08) Sponge

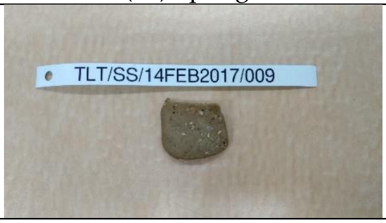

TLT/SS/14FEB2017/009
(09) Sponge

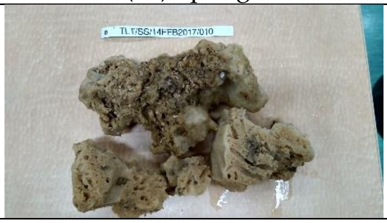

TLT/SS/14FEB2017/010
(10) Sponge

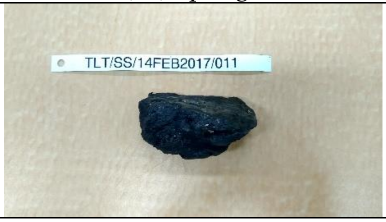

TLT/SS/14FEB2017/011
(11) Sediment

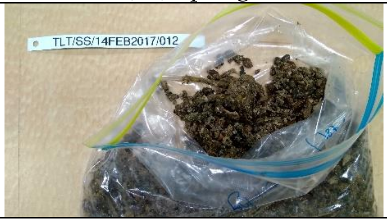

TLT/SS/14FEB2017/012
(12) Sediment

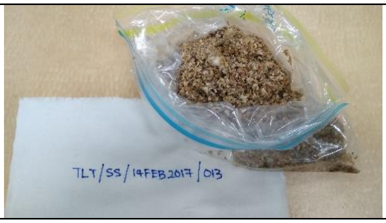

TLT/SS/14FEB2017/013
(13) Sediment

Table S2: Composition of the eight media and other reagents used for the isolation of culturable marine bacteria and other bioassays.

Media	Composition (g/L)
A1	2.0 Peptone, 10.0 Soluble Starch, 4.0 Yeast Extract, 18.0 Agar (Sigma-Aldrich), 1000 mL ASW, 0.015 Nalidixic Acid Sodium Salt, 0.05 Potassium Dichromate.
A2	1.0 Dipotassium Phosphate, 1.0 Calcium Chloride, 0.2 Iron Chloride, 1.0 Magnesium Sulphate, 0.1 Manganese Sulphate, 1.0 Potassium Nitrate, 0.01 Boric Acid, 0.02 Cobalt Chloride, 0.01 Copper Sulphate, 8.0 EDTA Iron (III) Sodium Salt, 0.005 Lithium Chloride, 0.1 Manganese Chloride, 0.02 Potassium Bromide, 0.02 Potassium Iodide, 0.01 Sodium Molybdate dehydrate, 0.005 Tin (II) Chloride dehydrate, 0.02 Zinc Chloride, 10.0 Agar (Sigma-Aldrich), 1000 mL ASW, 0.025 Cycloheximide, 0.015 Nalidixic Acid Sodium Salt.
A3	2.0 Peptone, 5.0 Soluble Starch, 2.0 Yeast Extract, 22.0 Red Sea Salt, 18.0 Agar (Sigma- Aldrich), 1000 mL Distilled Water, 0.015 Nalidixic Acid Sodium Salt, 0.05 Potassium Dichromate.
A4HT	1.0 Peptone, 5.0 Soluble Starch, 2.0 Yeast Extract, 18.0 Agar (Sigma-Aldrich), 500ml ASW, 500ml Distilled Water, 0.1 Cycloheximide, 0.015 Nalidixic Acid Sodium Salt.
A5	18.0 Agar (Sigma-Aldrich), 500ml ASW, 500 mL Distilled Water, 0.1 Cycloheximide, 0.015 Nalidixic Acid Sodium Salt.
MBA	55.1 g Marine Agar 2216 (BD Difco™), 1000 mL Distilled Water, 0.015 Nalidixic Acid Sodium Salt, 0.05 Potassium Dichromate.
SC	1.0 Casein Hydrolysate, 10.0 Soluble Starch, 15.0 Agar (Sigma-Aldrich), 1000 mL ASW, 0.08 Cycloheximide, 0.015 Nalidixic Acid Sodium Salt.
AIA	22.0 Actinomycetes Isolation Agar (Sigma- Aldrich), 5 mL Glycerol, 1000 mL ASW, 0.1 Cycloheximide, 0.015 Nalidixic Acid Sodium Salt.

Reagents	Composition (g/L)
ASW	33.33 Red Sea Salt
ABTGC	900 mL of BT-medium (1 Magnesium Chloride, 1 Calcium Chloride, 1 Iron (III) Chloride, 1000 mL Distilled Water)<br>100 mL of A10-medium (19.95 Ammonium Sulfate, 47.85 Sodium Phosphate dibasic, 29.94 Potassium dihydrogen Phosphate, 29.8 Sodium Chloride, 1000ml Distilled Water)<br>Supplemented with 10 mL of 20% Glucose and 10 mL of 20% Casamino Acids

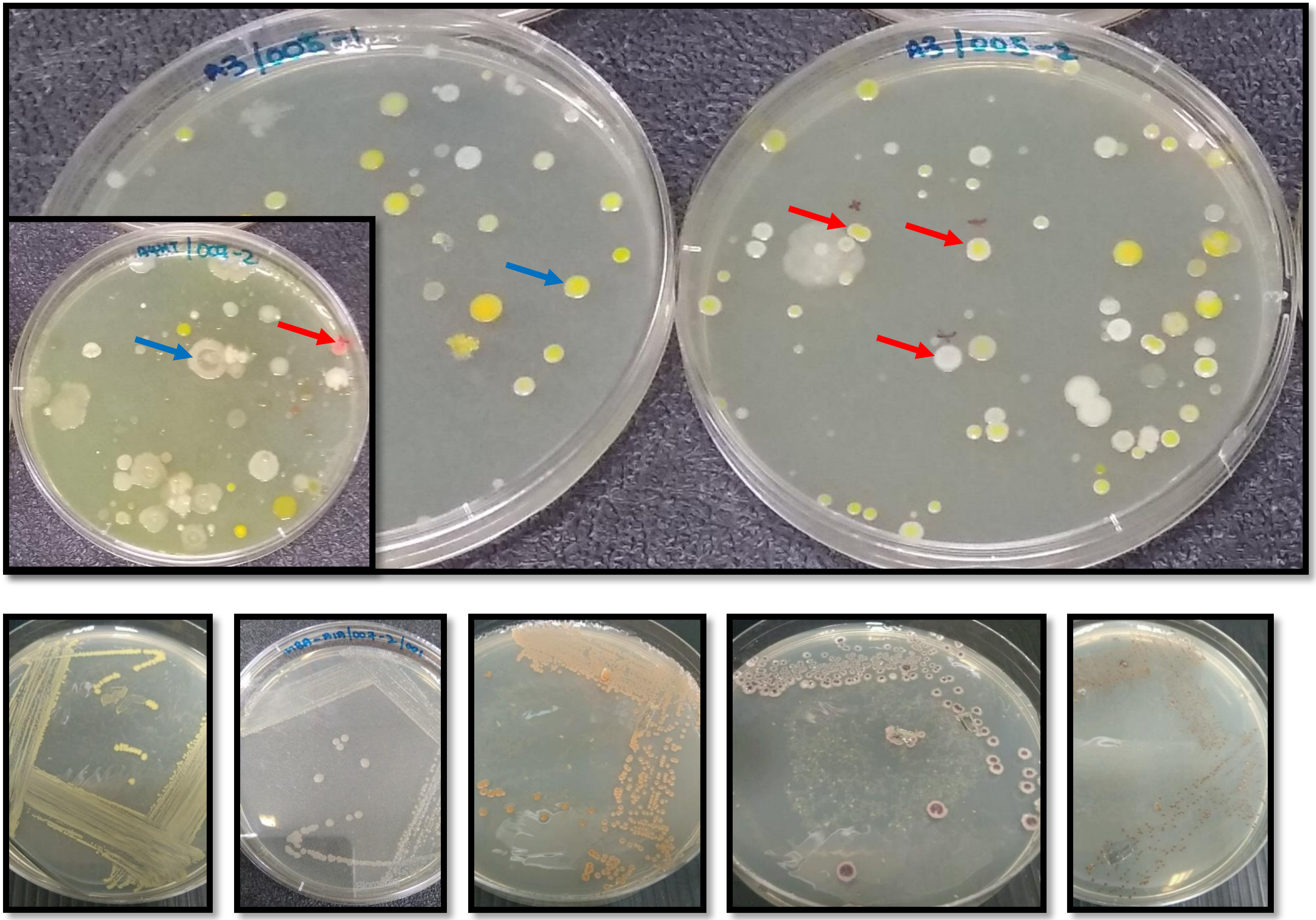

Figure S1. An example of colonies of interest (red arrow) that were isolated from the eight different isolation media. Colonies displaying interesting morphology, such as bright colors, matte textures, or unique colony shapes, were identified as our colonies of interest. Some of the other colonies (blue arrow) commonly appearing across the different isolation agar plates were also isolated as part of the colonies of interest to ensure that we are not bias in our colonies selection for the drug discovery process.

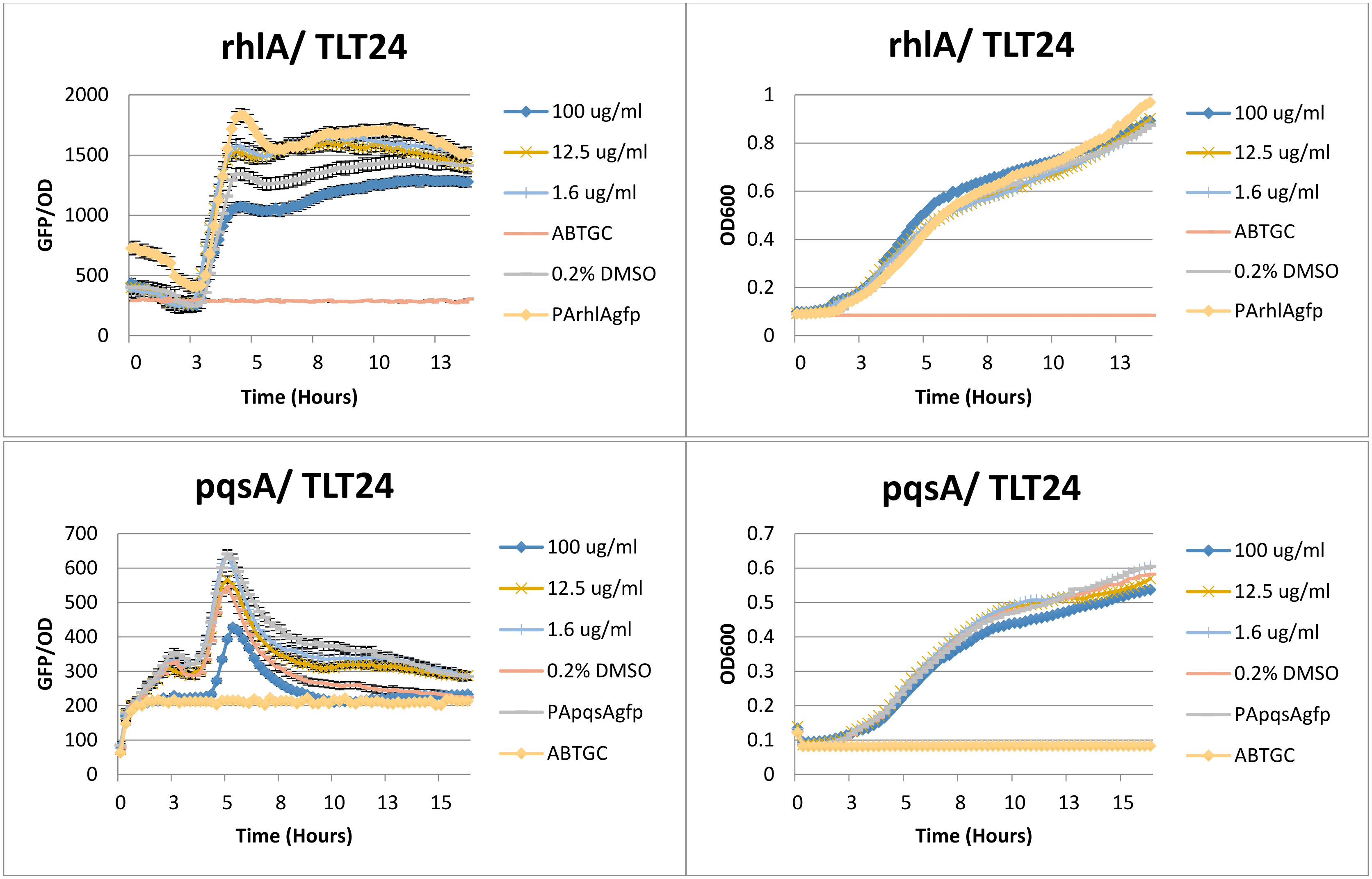

Figure S2. The dose-dependent inhibition curves (left graphs) from the *Pseudomonas aeruginosa* quorum sensing inhibition bioassay result incubated with crude extract prepared from TLT/SS/14FEB2017/005/A4HT-01/001 (#24) at the various concentration. The growth rates (right graphs) of the tested strain were not affected, showing that the inhibition effect observed is not due to any death of the tested strain. The experiments were conducted in triplicate and the average reading presented.

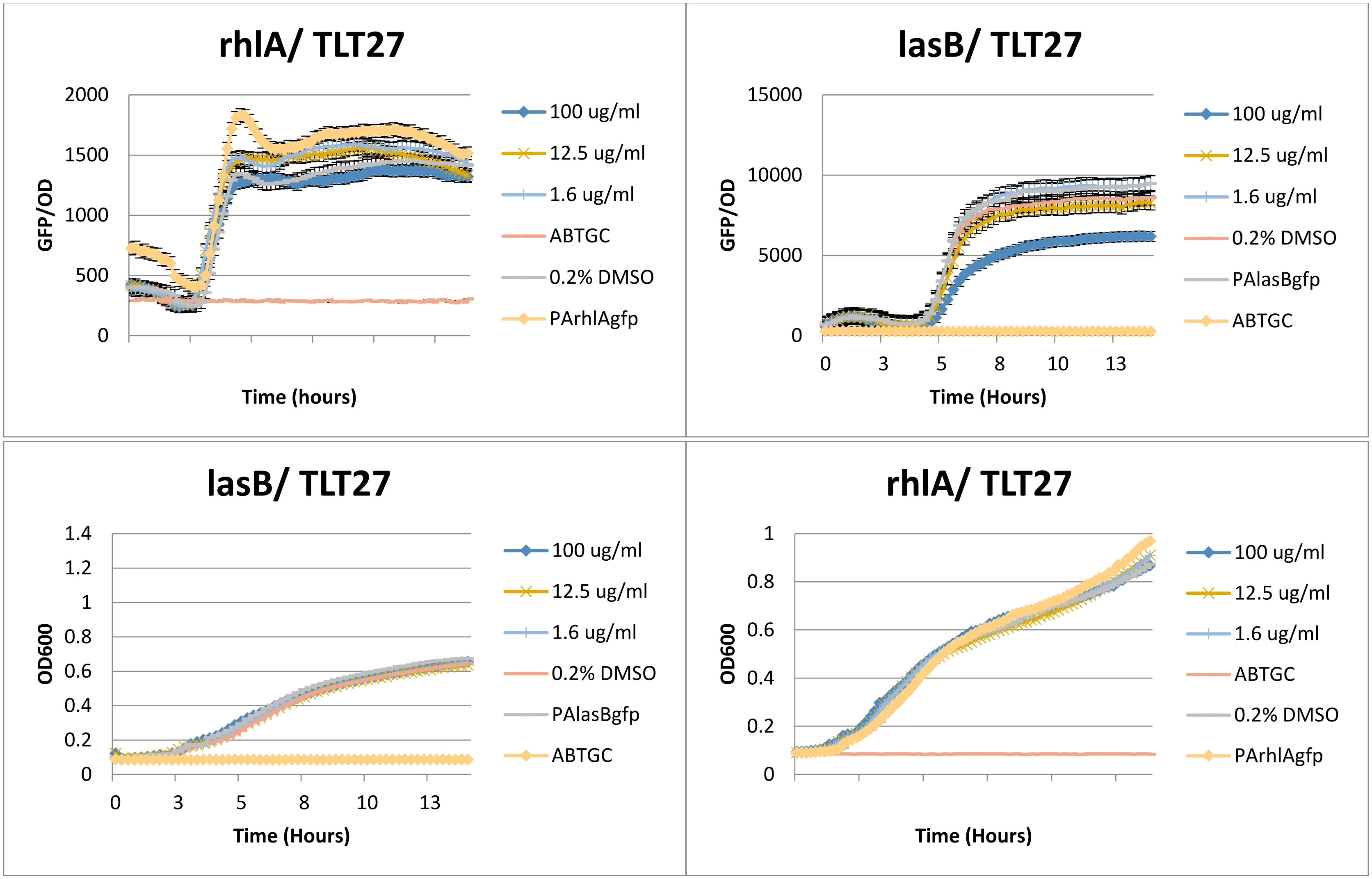
rhlA/ TLT27
GFP/OD
2000
1500
1000
500
0
Time (hours)
100 ug/ml
12.5 ug/ml
1.6 ug/ml
ABTGC
0.2% DMSO
PArhlAgfp
lasB/ TLT27
GFP/OD
15000
10000
5000
0
0
3
5
8
10
13
Time (Hours)
100 ug/ml
12.5 ug/ml
1.6 ug/ml
0.2% DMSO
PAlasBgfp
ABTGC
lasB/ TLT27
OD600
1.4
1.2
1
0.8
0.6
0.4
0.2
0
0
3
5
8
10
13
Time (Hours)
100 ug/ml
12.5 ug/ml
1.6 ug/ml
0.2% DMSO
PAlasBgfp
ABTGC
rhlA/ TLT27
OD600
1
0.8
0.6
0.4
0.2
0
Time (Hours)
100 ug/ml
12.5 ug/ml
1.6 ug/ml
ABTGC
0.2% DMSO
PArhlAgfp

Figure S3. *Cont.*

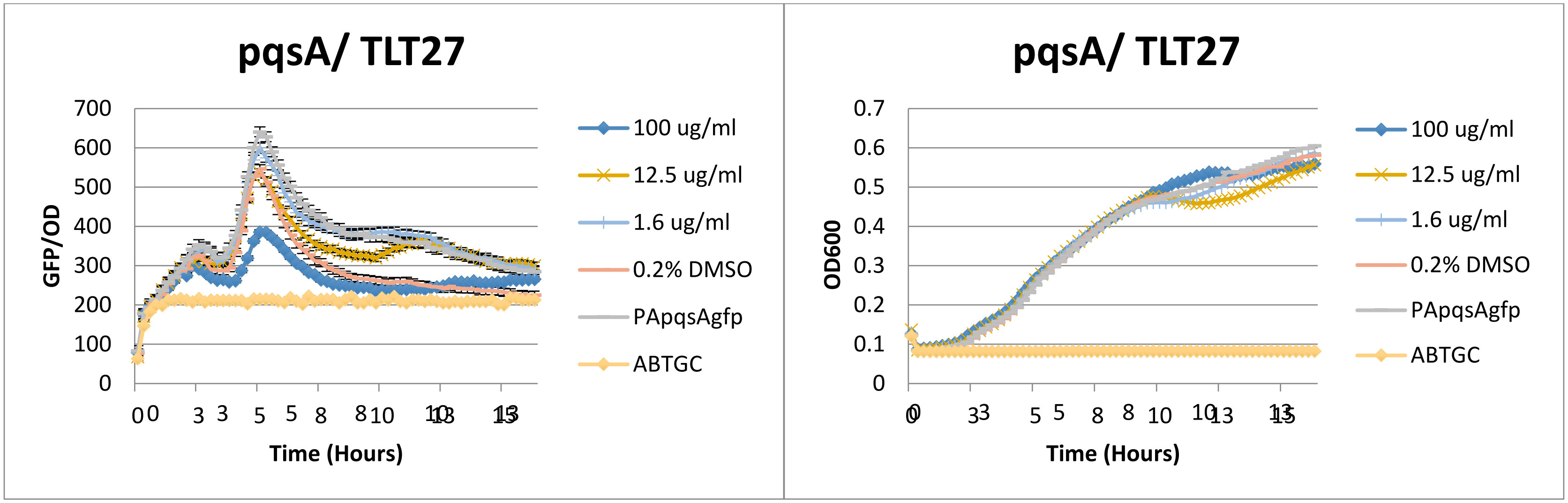

Figure S3. The dose-dependent inhibition curves (left graphs) from the *Pseudomonas aeruginosa* quorum sensing inhibition bioassay result incubated with crude extract prepared from TLT/SS/14FEB2017/005/A5-01/001 (#27) at the various concentration. The growth rates (right graphs) of the tested strain were not affected, showing that the inhibition effect observed is not due to any death of the tested strain. The experiments were conducted in triplicate and the average reading presented.

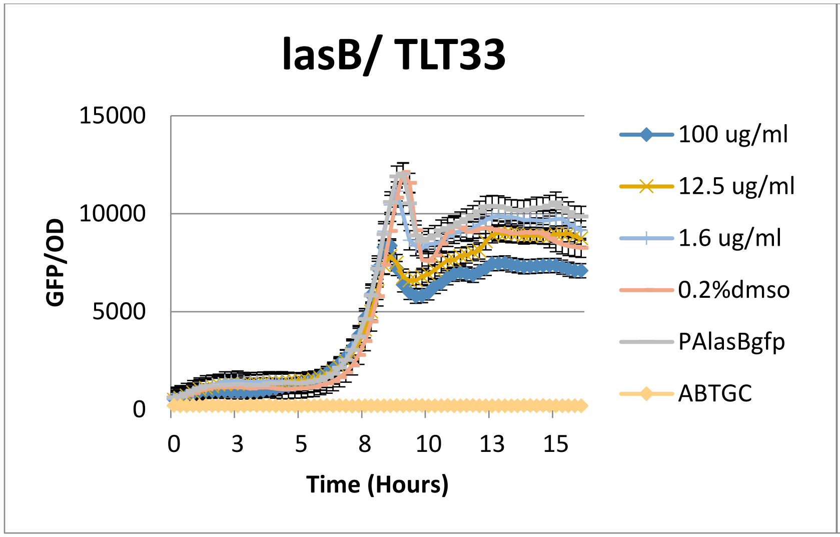
lasB/ TLT33
GFP/OD
15000
10000
5000
0
0
3
5
8
10
13
15
Time (Hours)
100 ug/ml
12.5 ug/ml
1.6 ug/ml
0.2%dmso
PAlasBgfp
ABTGC

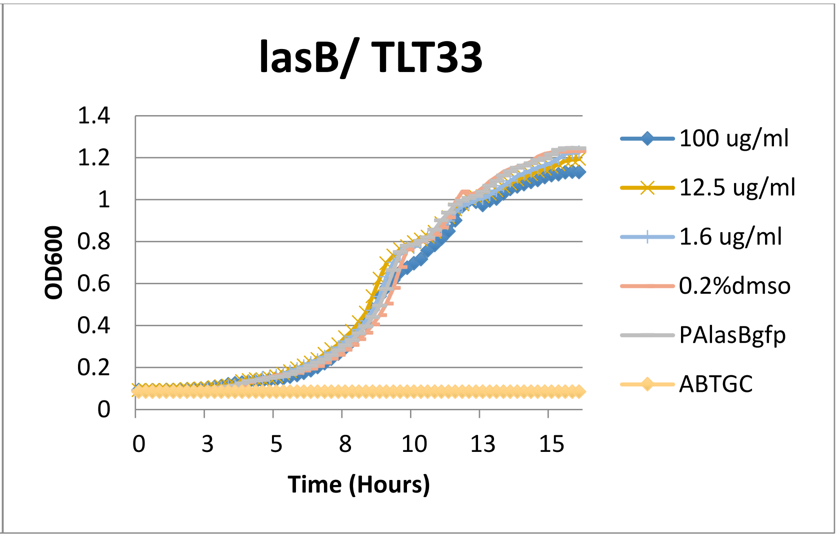
lasB/ TLT33
OD600
1.4
1.2
1
0.8
0.6
0.4
0.2
0
0
3
5
8
10
13
15
Time (Hours)
100 ug/ml
12.5 ug/ml
1.6 ug/ml
0.2%dmso
PAlasBgfp
ABTGC

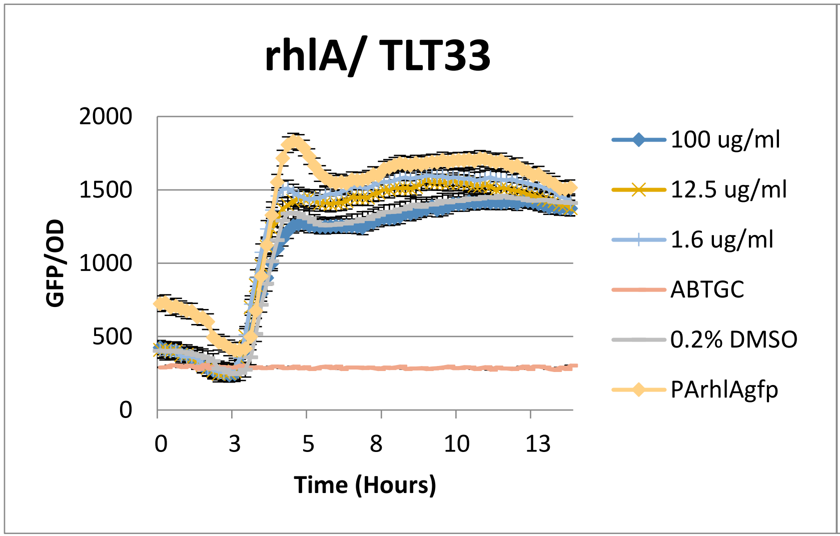
rhlA/ TLT33
GFP/OD
2000
1500
1000
500
0
0
3
5
8
10
13
Time (Hours)
100 ug/ml
12.5 ug/ml
1.6 ug/ml
ABTGC
0.2% DMSO
PArhlAgfp

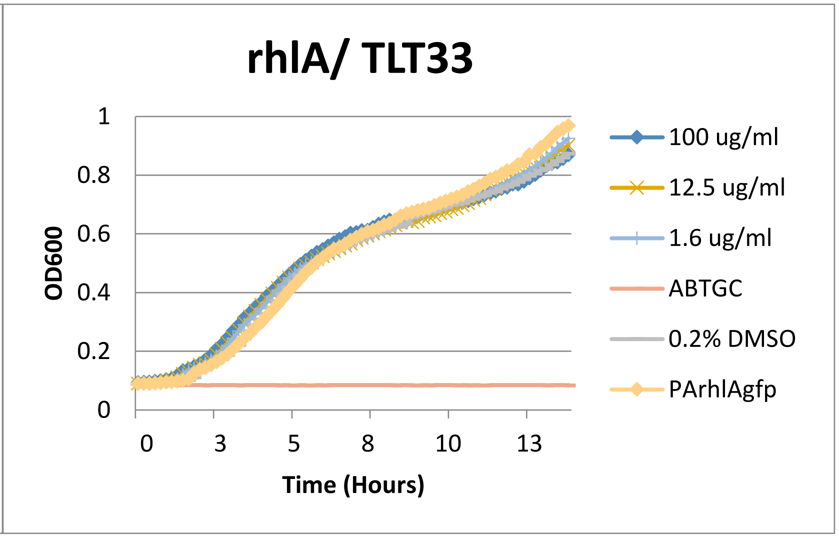
rhlA/ TLT33
OD600
1
0.8
0.6
0.4
0.2
0
0
3
5
8
10
13
Time (Hours)
100 ug/ml
12.5 ug/ml
1.6 ug/ml
ABTGC
0.2% DMSO
PArhlAgfp

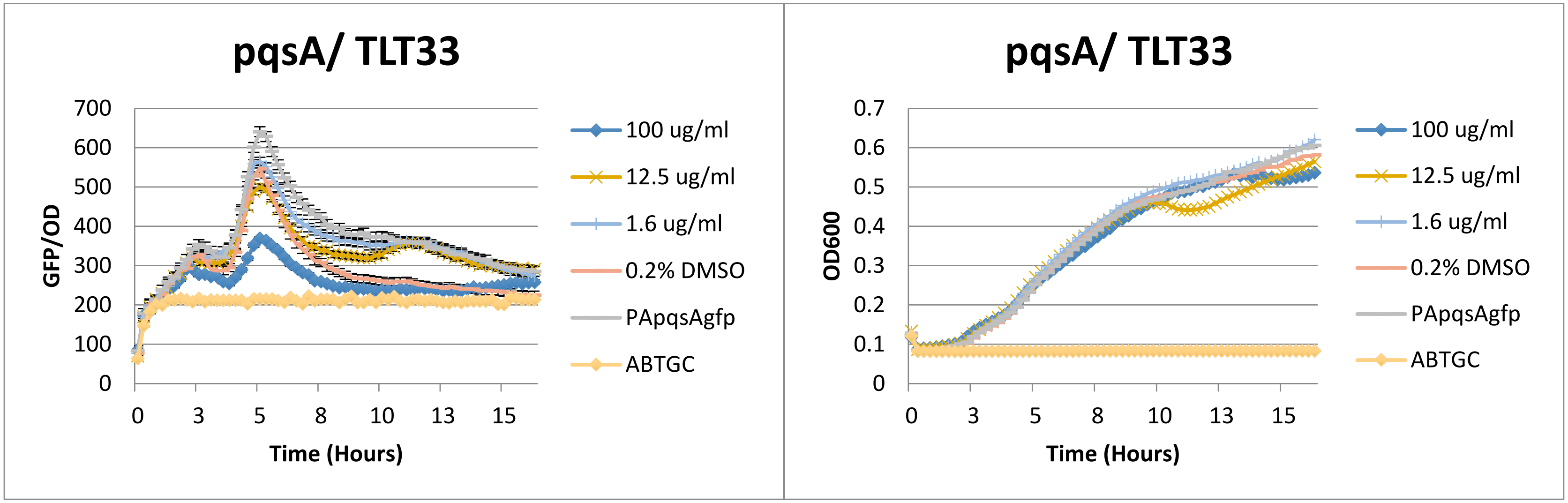

Figure S4. The dose-dependent inhibition curves (left graphs) from the *Pseudomonas aeruginosa* quorum sensing inhibition bioassay result incubated with crude extract prepared from TLT/SS/14FEB2017/005/MBA-02/004 (#33) at the various concentration. The growth rates (right graphs) of the tested strain were not affected, showing that the inhibition effect observed is not due to any death of the tested strain. The experiments were conducted in triplicate and the average reading presented.

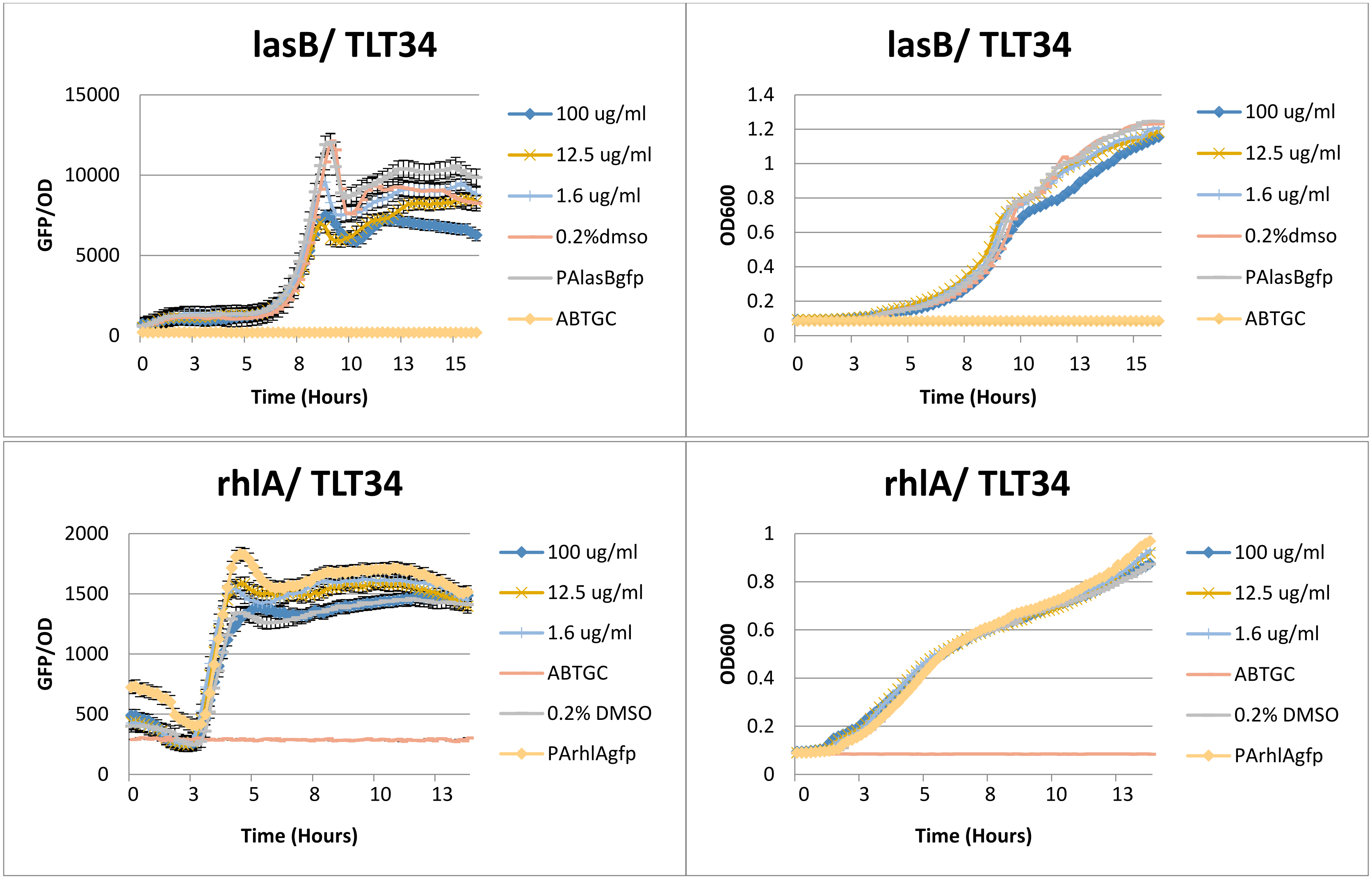
lasB/ TLT34
GFP/OD
15000
10000
5000
0
0 3 5 8 10 13 15
Time (Hours)
100 ug/ml
12.5 ug/ml
1.6 ug/ml
0.2%dmso
PAlasBgfp
ABTGC
lasB/ TLT34
OD600
1.4
1.2
1
0.8
0.6
0.4
0.2
0
0 3 5 8 10 13 15
Time (Hours)
100 ug/ml
12.5 ug/ml
1.6 ug/ml
0.2%dmso
PAlasBgfp
ABTGC
rhlA/ TLT34
GFP/OD
2000
1500
1000
500
0
0 3 5 8 10 13
Time (Hours)
100 ug/ml
12.5 ug/ml
1.6 ug/ml
ABTGC
0.2% DMSO
PArhlAgfp
rhlA/ TLT34
OD600
1
0.8
0.6
0.4
0.2
0
0 3 5 8 10 13
Time (Hours)
100 ug/ml
12.5 ug/ml
1.6 ug/ml
ABTGC
0.2% DMSO
PArhlAgfp

Figure S5. *Cont.*

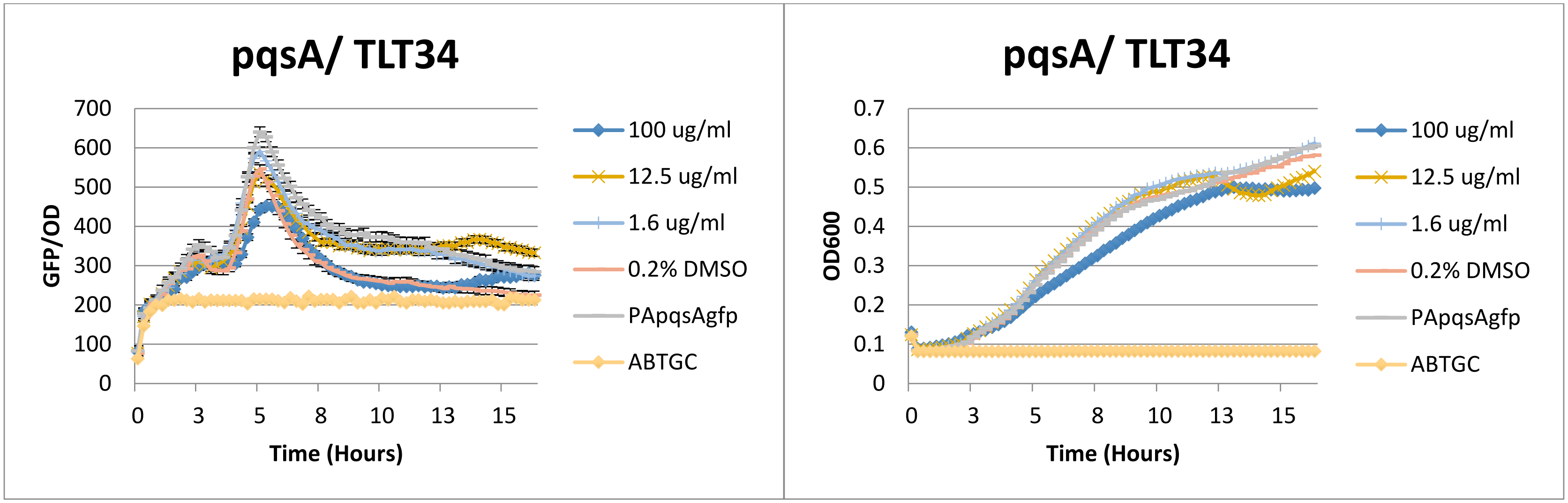

Figure S5. The dose-dependent inhibition curves (left graphs) from the *Pseudomonas aeruginosa* quorum sensing inhibition bioassay result incubated with crude extract prepared from TLT/SS/14FEB2017/005/SC-01/001 (#34) at the various concentration. The growth rates (right graphs) of the tested strain were not affected, showing that the inhibition effect observed is not due to any death of the tested strain. The experiments were conducted in triplicate and the average reading presented.

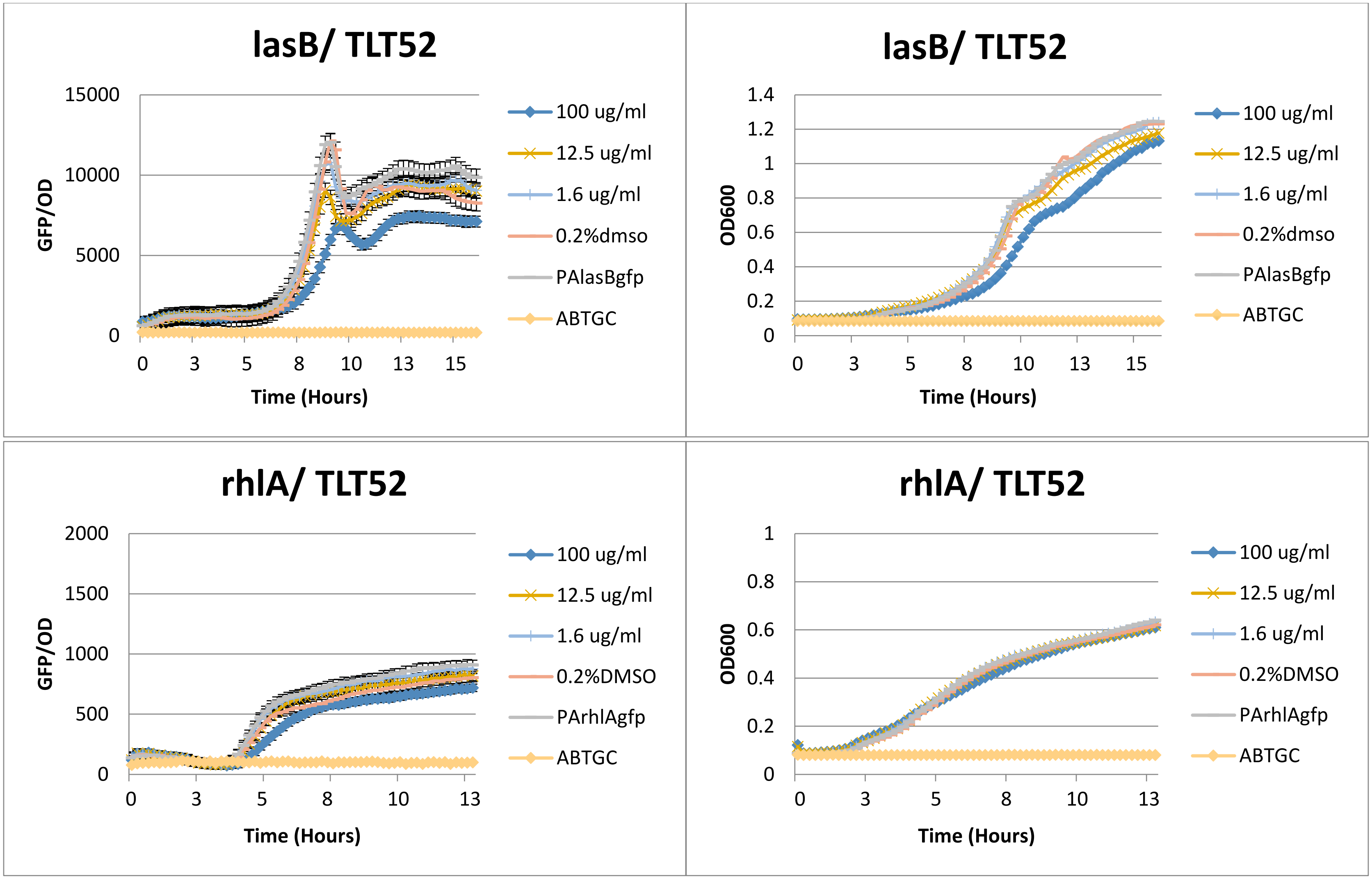

Figure S6. The dose-dependent inhibition curves (left graphs) from the *Pseudomonas aeruginosa* quorum sensing inhibition bioassay result incubated with crude extract prepared from TLT/SS/14FEB2017/007/AIA-02/001 (#52) at the various concentration. The growth rates (right graphs) of the tested strain were not affected, showing that the inhibition effect observed is not due to any death of the tested strain. The experiments were conducted in triplicate and the average reading presented.

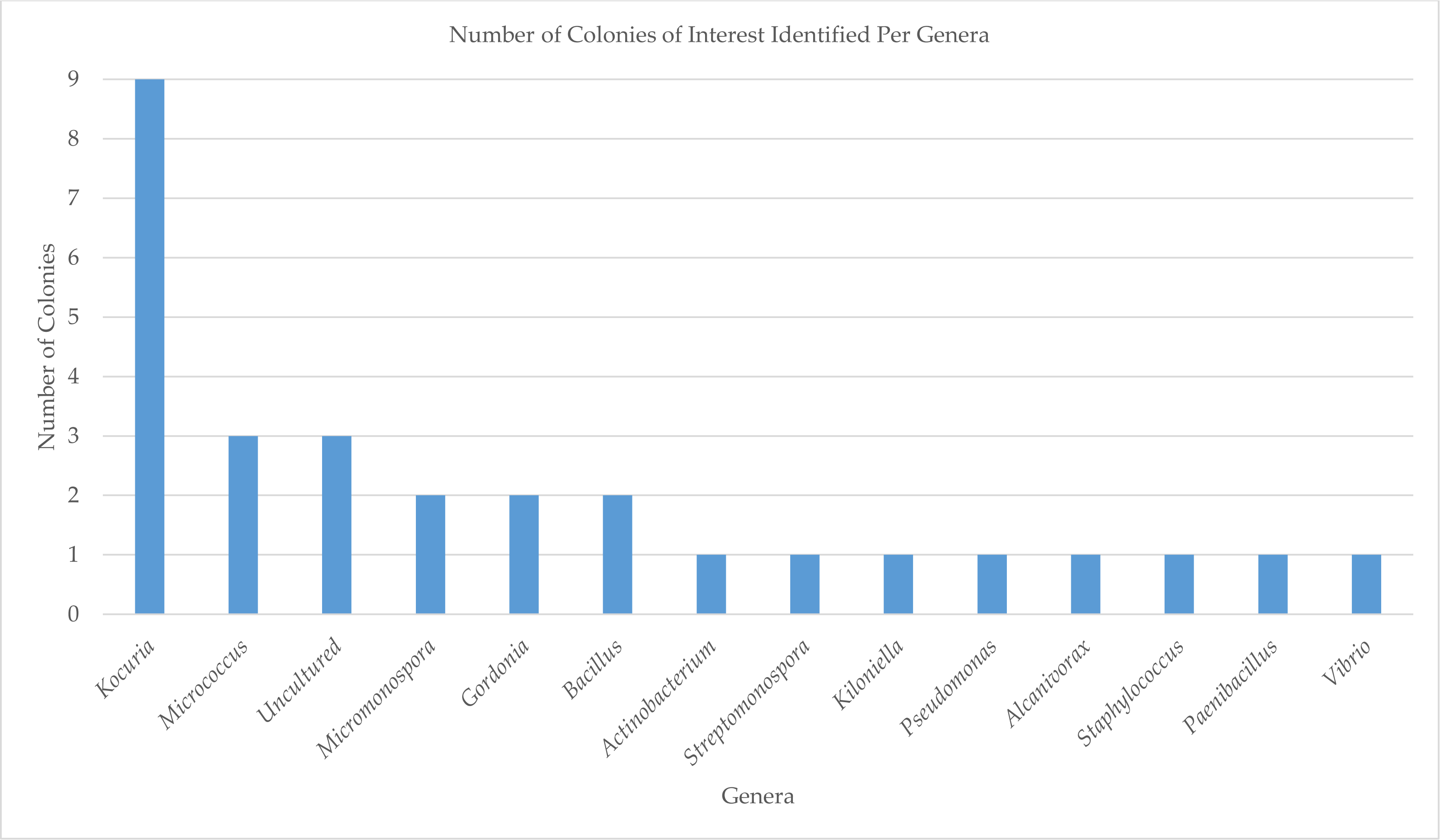

Figure S7. Number of colonies of interest identified per genera based on the 16S rRNA gene sequencing result.
